# Chronic Ketogenic Low Carbohydrate High Fat Diet Has Minimal Effects on Acid–Base Status in Elite Athletes

**DOI:** 10.3390/nu10020236

**Published:** 2018-02-18

**Authors:** Amelia J. Carr, Avish P. Sharma, Megan L. Ross, Marijke Welvaert, Gary J. Slater, Louise M. Burke

**Affiliations:** 1 Centre for Sport Research, Deakin University, Burwood, VIC 3125, Australia; 2 Physiology, Australian Institute of Sport, Bruce, ACT 2617, Australia; avish.sharma@ausport.gov.au; 3 Research Institute for Sport and Exercise, University of Canberra, Belconnen, ACT 2616, Australia; marijke.welvaert@ausport.gov.au; 4 Sports Nutrition, Australian Institute of Sport, Bruce, ACT 2617, Australia; meg.ross@ausport.gov.au (M.L.R.); louise.burke@ausport.gov.au (L.M.B.); 5 Mary MacKillop Institute for Health Research, Australian Catholic University, Melbourne, VIC 3000, Australia; 6 Innovation, Research and Development, Australian Institute of Sport, Bruce, ACT 2617, Australia; 7 School of Health and Sport Sciences, University of the Sunshine Coast, Maroochydore, QLD 4558, Australia; gslater@usc.edu.au

**Keywords:** dietary interventions, periodized carbohydrate diet, fat adaptation, keto-adaptation

## Abstract

Although short (up to 3 days) exposure to major shifts in macronutrient intake appears to alter acid–base status, the effects of sustained (>1 week) interventions in elite athletes has not been determined. Using a non-randomized, parallel design, we examined the effect of adaptations to 21 days of a ketogenic low carbohydrate high fat (LCHF) or periodized carbohydrate (PCHO) diet on pre- and post-exercise blood pH, and concentrations of bicarbonate [HCO_3_^−^] and lactate [La^−^] in comparison to a high carbohydrate (HCHO) control. Twenty-four (17 male and 7 female) elite-level race walkers completed 21 days of either LCHF (*n* = 9), PCHO (*n* = 7), or HCHO (*n* = 8) under controlled diet and training conditions. At baseline and post-intervention, blood pH, blood [HCO_3_^−^], and blood [La^−^] were measured before and after a graded exercise test. Net endogenous acid production (NEAP) over the previous 48–72 h was also calculated from monitored dietary intake. LCHF was not associated with significant differences in blood pH, [HCO_3_^−^], or [La^−^], compared with the HCHO diet pre- or post-exercise, despite a significantly higher NEAP (mEq·day^−1^) (95% CI = (10.44; 36.04)). Our results indicate that chronic dietary interventions are unlikely to influence acid–base status in elite athletes, which may be due to pre-existing training adaptations, such as an enhanced buffering capacity, or the actions of respiratory and renal pathways, which have a greater influence on regulation of acid–base status than nutritional intake.

## 1. Introduction

Low carbohydrate high fat (LCHF) diets have previously been implemented in the context of epilepsy treatment [1,2] and as a weight loss strategy [3,4]. More recently, however, there has been a re-emergence of interest in their potential role in sports nutrition, with claims that adaptation to restricted carbohydrate (CHO) intake and high levels of circulating ketone bodies by trained individuals achieves significant changes in substrate utilization during sub-maximal exercise, to shift reliance from glycogen utilization to the relatively unlimited stores of body fat [5]. Indeed, both early [6] and more recent [7,8,9] studies have shown that sustained (3 weeks to several years) exposure to such a diet causes these robust shifts in exercise fuel use. However, beneficial effects on performance remain unsubstantiated, with reports of maintained capacity for submaximal cycling under fasted conditions in well-trained cyclists [6], but a reduction in exercise economy and failure to improve 10,000 m race performance following a block of intensified training in elite race walkers [9], in comparison to a more traditional diet providing high CHO availability [10]. A third approach to nutrition support for endurance sport is the periodized CHO diet, which integrates strategies to achieve high CHO availability to support key training sessions with protocols for low CHO availability to enhance adaptive responses to selected lower intensity sessions [11]. This redistribution of CHO intake to target the individualized goals of each training session has been shown to alter substrate utilization during submaximal exercise [12], and to produce performance benefits in sub-elite [11,13], but not elite [9,14] athletes. Thus, it appears that sports performance is determined by factors other than a simple change in substrate utilization. 

One of the less well-studied effects of sustained manipulations of the macronutrient composition of the diet is the alteration in acid–base status. Diets high in fat can increase blood acidity, attributed to the stimulation of lipolysis, and the release of acidic ketone bodies [4]. A small number of studies have investigated the effects of short-term dietary modifications on acid–base status and exercise capacity in healthy but essentially untrained individuals [15,16,17,18]. For example, measurement of pre-exercise blood pH and bicarbonate concentrations after an overnight fast showed that three days of a low (<10% energy intake) CHO diet was associated with a reduction in blood alkalinity, compared with a high (>65% energy) CHO diet [15]. Other studies by the same group confirmed the effect of various macronutrient concentrations. After a three-day high-protein (24% total energy intake), high fat diet (73% total energy intake) [17], and a three-day low-carbohydrate diet (4% total energy intake) [16], pre- and post-exercise blood pH and blood bicarbonate concentrations decreased, compared with a high carbohydrate diet. While these results indicate modifications of acid–base status after acute alterations in dietary macronutrient intake, and potentially a separate and additional contribution to changes in exercise capacity, the effects have yet to be determined for sustained dietary interventions, particularly in athletic populations. 

Acid–base status has primarily been investigated in terms of the deleterious health implications of metabolic acidosis [19,20,21,22]. Damage to bone and muscle can occur when additional calcium is excreted in response to the excess of protons, and adverse effects, such as kidney stones, can be caused by a reduced urine pH, a further compensatory mechanism against acidosis [23,24]. Small changes to acid–base status place substantial stress on the body’s buffering mechanisms [23,25], and one contributing factor is the composition of the diet [26]. It is acknowledged that normal metabolism incorporates many reactions that produce and consume acids and bases, and that acid–base status is routinely corrected by a tightly controlled acid–base regulatory system. The discrepancy between the acid and base forming reactions due to dietary intake is, however, the primary contributing factor toward net endogenous acid production (NEAP) [25].

NEAP can be calculated via validated equations [26,27]. One widely used method focuses on the consumption of protein and potassium, quantified over a 24 h period [26]; since protein is a sulfuric acid precursor, and potassium ingestion results in bicarbonate formation, the two dietary components have a substantial influence on acid production [26]. Another more comprehensive estimation of acid–base status involves more variables, including the intake of protein and specified micronutrients (potassium, phosphate, magnesium, and calcium) over 24 h, body surface area (which affects acid excretion rates), and established intestinal absorption rates of different dietary components [27]. Several previous studies have estimated acid production due to short-term (7 days or less) dietary interventions [15,16,17,18,28,29] using a similar principle to the two approaches described, but NEAP has not been calculated for sustained dietary interventions. NEAP calculations could potentially provide an indication of the effect of different dietary regimes on acid–base status, particularly when combined with direct measures of acid–base status, such as blood pH. There are potential implications for athletes’ performance, given that an increased alkalosis or buffering capacity can improve performance, and a reduction in blood or muscle pH can be detrimental to high-intensity exercise performance [30,31]. Furthermore, differences in acid–base status between athletes and untrained individuals have been reported, associated with differences in buffering capacity [32,33].

The goal of the current study was to fill gaps in our current knowledge on the longer-term changes in acid–base status associated with diets that have been shown to alter blood pH and bicarbonate concentrations when followed for short periods. In particular, we wanted to investigate the effects of adaptation to a sustained ketogenic diet or periodized carbohydrate availability on such markers before and after exercise in elite athletes, and whether NEAP calculations of endogenous acid production were able to predict or explain any changes. Therefore, our aims were to determine the effect of adaptations to two sustained dietary interventions of current interest to endurance athletes (a LCHF diet and a periodized carbohydrate availability diet [9]) on blood bicarbonate concentration, blood pH, and blood lactate concentration (pre and post exercise), and net endogenous acid production (NEAP).

## 2. Materials and Methods

**Figure 1:**
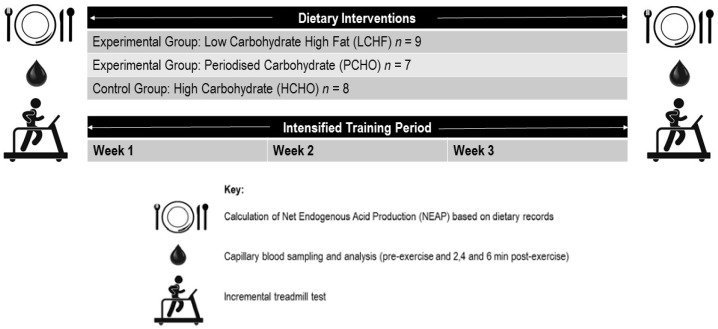
Overview of testing conducted in the study. All testing was conducted at baseline (prior to any intervention), and post-testing (after a supervised three-week training and dietary intervention).

A non-randomized parallel groups study design was used to determine the effect of three sustained (3 week) dietary interventions (a control high CHO diet—HCHO; low carbohydrate high fat diet—LCHF, and periodized carbohydrate availability diet—PCHO) on acid base balance, at rest, and following a graded exercise test in national and international level race walkers (Figure 1). Acid–base status was measured via capillary blood samples, which were analyzed for blood pH, blood bicarbonate [HCO_3_^−^] concentration, and blood lactate [La^−^] concentration. Furthermore, calculations were undertaken to determine net endogenous acid production (NEAP) of the diets from dietary records kept over the 48 h prior to each exercise test. These measurements were conducted at two timepoints: baseline (prior to any intervention and with participants following a self-chosen diet), and post-testing (after a three-week dietary and training intervention, during which all food was provided to participants and consumed under supervision). The study was part of a larger research project, conducted during a residential training camp held throughout January and February 2017 at the Australian Institute of Sport (AIS; Canberra, Australian Capital Territory, Australia). All participants gave their written informed consent for inclusion, prior to their participation in the study. The study was conducted in accordance with the Declaration of Helsinki, and the protocol was approved by the AIS Ethics Committee (Project Approval Code: 20161201).

### 2.1. Participants

Twenty-eight participants volunteered for this study, with twenty-four participants completing all testing (Table 1). Two males and one female were unable to complete the requirements due to injury, and one male was unable to provide a full data set for the baseline testing due to their delayed arrival at the training camp. The cohort consisted of elite race walkers, competitive at international or national level (mean ± SD International Association of Athletics Federations points = 1130 ± 52) [34] who participated in a large dietary intervention study conducted at the Australian Institute of Sport with the support of Athletics Australia (further details are provided by Burke et al., submitted for publication).The study included participants from Australia, New Zealand, Poland, United Kingdom, South Africa, Lithuania, Canada, United States of America, Chile, Hungary, Japan, and Spain. A sample size estimation performed for the larger study, based upon outcome measures associated with performance and substrate utilization from recent research from our group with a similar cohort, indicated that eight participants per group would be required to detect physiological differences [9]. Participants were assigned to one of the two dietary intervention groups (LCHF or PCHO), or the HCHO control group, according to their preferred dietary intervention. 

### 2.2. Training Intervention

Athletes followed a supervised and monitored training program across a three-week period of intensified training (Table 2). Of the ~12 weekly training sessions, six involved mandatory and supervised group sessions of race walking. There were two long sessions of ≥20 km, a track session involving sustained high-intensity 1-km repetitions on a 6-min cycle, a 14-km hill training session (change in elevation ~285 m), and two low–moderate intensity “recovery” sessions, while two resistance training and three hydrotherapy sessions were completed. The athletes could modify their remaining sessions, undertaking them as additional race walking sessions, or substituting with cross training, such as swimming or cycling. All athletes recorded their daily training in standardized training logs.

### 2.3. Dietary Intervention

All meals were prepared by chefs, according to standardized recipes, and were consumed by participants in a group environment, under the supervision of registered sports dietitians, according to a previously established practice [32]. For baseline testing, participants were able to choose freely from items provided as a buffet-style menu at the AIS Dining Hall, and were assisted to weigh and record all of their food intake during this period, using calibrated food scales (SJ-5001HS, A&D Weighing, Australia). Once the dietary intervention commenced, participants were provided with all meals and snacks according to their intervention group (PCHO or LCHF, or HCHO) with menus being individualized to BM and training load (to provide an energy availability of ~40 kcal·kg^−1^ lean BM; LBM), and specific dietary requirements including food allergies, intolerances, and dietary preferences. Each athlete could request an increase or decrease in the quantities of foods or drinks provided according to hunger, changes in training load, or fluctuations in BM. Such variations were accommodated by adhering to the macronutrient composition of that individual’s treatment group. During this period, all food intake was weighed prior to consumption, with allowances made for unfinished portions or additional snacks chosen from a menu-specific list, as per food diary logs. Energy and nutrient intake provided by the diets was calculated from a food analysis database specific to Australian foods (Foodworks Version 9, Highgate Hill, Australia) by the same registered dietitian. Full details of the methodology for creating, providing, and recording food intake can be found elsewhere [35].

The three dietary interventions were chosen to represent different approaches to the support of high volume training programs [9].

- HCHO: traditional sports nutrition guidelines promoting high carbohydrate availability for all training sessions: CHO: ~8 g·kg^−1^ BM, 60–65% energy intake; protein: ~1.8 g·kg^−1^·day^−1^ 15–20% energy; fat: ~20% energy intake [10].
- PCHO: contemporary approach to sports nutrition, with same energy and macronutrient composition as HCHO, but manipulated across and between days to provide high CHO availability for key training sessions and low CHO availability for other sessions [11,36].
- LCHF: popular ketogenic low CHO high fat diet: CHO: <50 g·day^−1^, protein: ~1.8 g·kg^−1^·day^−1^ 15–20% protein, and 75–80% fat [6,34].

### 2.4. Exercise Testing

All participants completed a graded maximal exercise test at the baseline and post-testing timepoints, under controlled laboratory conditions. All participants conducted each test in a fasted state. The test was performed on a custom-built, motorized treadmill (Australian Institute of Sport, Canberra, Australia). The test was comprised of four submaximal stages (for determination of submaximal VO_2_ and walking economy). Each stage, three minutes in length, was immediately followed by an incremental ramp to exhaustion for determination of VO_2_peak. As such, the total test duration was approximately 13 to 20 min, depending on a participant’s time to exhaustion (TTE). The treadmill velocity for the first stage was dependent on each participants’ most recent 10 km race time (9–12 km·h^−1^), at 0% gradient, with the velocity being increased by 1 km·h^−1^ with each subsequent stage. After each stage, a small (5 microlitres; μL) capillary blood sample was taken from the fingertip, to measure blood [La^−^] (Lactate Pro, Arkray, Kyoto, Japan). Immediately following the completion of the fourth submaximal stage (approximately equivalent to 20 km race-walk speed), the gradient of the treadmill was increased by 0.5 degrees every 30 s, until the participant reached volitional exhaustion. Heart rate (Polar heart rate monitor, Polar Electro, Kempele, Finland) was measured throughout the test. Expired ventilation samples were collected continuously throughout the test, using a custom-built open-circuit indirect calorimetry system (Australian Institute of Sport, Canberra, Australia). The system was calibrated prior to each test [37].

Prior to starting the maximal exercise test, 100 μL of capillary blood was collected from the fingertip. The hand was first immersed in warm water for ~1 min to increase blood flow to the area, and then the fingertip was pierced using a sterile retractable lancet (Accu-Check, Roche, Sydney, Australia). Blood samples were immediately analyzed for blood pH, blood [HCO_3_^−^], and blood [La^−^] using a portable blood-gas analyzer (i-STAT, Abbott, Chicago, IL, USA), which was calibrated prior to testing, in accordance with manufacturers guidelines. The blood sampling and analysis procedure was repeated 2, 4, and 6 min after the completion of the maximal exercise test.

### 2.5. NEAP Calculations

The NEAP of dietary intake preceding the baseline and post-testing timepoints was calculated from the participant’s dietary records. Mean values were used to account for any fluctuation in dietary intake across days, and incorporated as much dietary information as was possible. Therefore, for baseline testing, values were determined from the mean intake recorded for the previous 48 h (the time between participants arriving at the training camp and the commencement of their dietary intervention), and post-testing calculations were taken from mean intake over the previous 72 h (the final three days of their supervised dietary intervention). Several published algorithms were used to estimate NEAP. These included two equations established by Frassetto et al. [26] which use daily protein and potassium intake, and one further equation developed by Remer and Manz [27], which incorporates daily protein, phosphorus, potassium, magnesium, and calcium intake, as well as anthropometric measurements. The three equations used are listed below.

1. Estimated NEAP (mEq·day^−1^) = (0.91 × protein (g·day^−1^)) − (0.57 × potassium (mEq·day^−1^)) + 21 [26] (NEAP_F1_).
2. Estimated NEAP (mEq·day^−1^) = (54.5 × protein (g·day^−1^)/potassium (mEq·day^−1^)) − 10.2 [26] (NEAP_F2_).
3. Estimated NEAP (mEq·day^−1^) = Potential Renal Acid Load (PRAL) (mEq·day^−1^) + Estimated Urinary Organic Anions (OA_est_) [38]. Within Equation (3), PRAL is calculated as: (mEq·day^−1^) = 0.488 × protein (g·day^−1^) + 0.037 × phosphorous (mg·day^−1^) − 0.021 × potassium (mg·day^−1^) – 0.026 × magnesium (mg·day^−1^) – 0.013 × calcium (mg·day^−1^), whereby: OA_est_ (mEq·day^−1^) = (0.007184 × height^0.725^ × mass^0.425^) × (41/1.73) (NEAP_R_) [27].

### 2.6. Statistical Analysis

The data were analyzed with a general linear mixed model using the R package lme4 (R Core Team, Vienna, Austria) [39]. A random intercept for participants included interindividual homogeneity. All models were estimated using restricted maximum likelihood. Visual inspection of residual plots did not reveal any obvious deviations from homoscedasticity or normality. *p*-Values were obtained using Type II Wald F tests with Kenward-Roger degrees of freedom as implemented in the R package car [40]. Results for all variables (blood pH, blood [HCO_3_^−^], blood [La^−^], NEAP) are reported as mean estimates and 95% confidence intervals. Initial models included all possible interactions, but non-significant interaction terms were dropped from the models for ease of interpretation.

## 3. Results

### 3.1. Participants

Participants’ pre-intervention VO_2_max was 57.6 ± 4.6 mL·kg·min^−1^ BM for HCHO, 58.1 ± 3.3 mL for PCHO and 61.1 ± 5.3 for LCHF. Post-intervention, VO_2_max was 58.3 ± 4.1, 60.2 ± 3.8, and 63.4 ± 4.1 mL·kg·min^−1^ BM, respectively. Within each of the three groups, there was a significant increase in VO_2_max compared with baseline (*p* < 0.05). 

### 3.2. Blood pH

**Figure 2:**
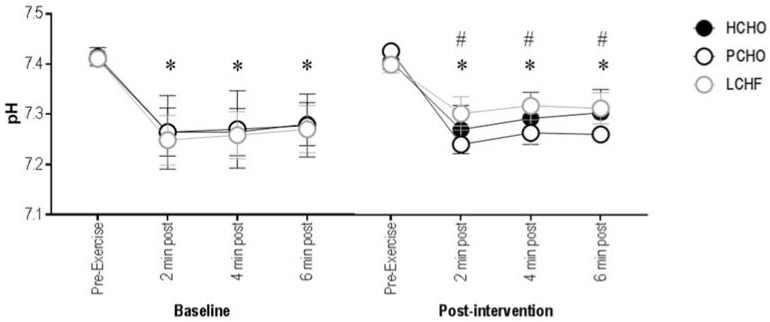
Blood pH for the high carbohydrate (HCHO), periodized carbohydrate (PCHO), and low carbohydrate high fat (LCHF) groups for baseline and post-intervention, at pre-exercise, plus 2, 4, and 6 min post-test. * Significantly different to pre-exercise (*p* < 0.05). # PCHO significantly different to LCHF (*p* < 0.05).

At baseline, there were no differences in blood pH between HCHO, PCHO, and LCHF, at any timepoint (F(2,20.97) = 0.86; *p* = 0.44), and as expected, there was a significant decrease in blood pH from pre-exercise to all post-exercise timepoints for all groups (F(1,144.10) = 8.64; *p* = 0.003). Post-intervention, there were no significant differences between groups at the pre-exercise collection (95% CI = (−0.03; 0.05) and (−0.01; 0.07) for HCHO and PCHO, compared to LCHF, respectively), but at the 2 min post-exercise timepoint, pH was significantly lower for PCHO compared with LCHF (95% CI = (−0.16; −0.04)), with no significant difference between HCHO and LCHF (95% CI = (−0.11; 0.01)). Similarly, at 4 min and 6 min post-exercise, pH was significantly lower for PCHO compared to LCHF (95% CI = (−0.15; −0.04) and (−0.15; −0.03), respectively), with no significant difference between HCHO and LCHF (95% CI = (−0.10; 0.02) and (−0.08; 0.04), respectively) (Figure 2).

### 3.3. Blood Bicarbonate Concentration

**Figure 3:**
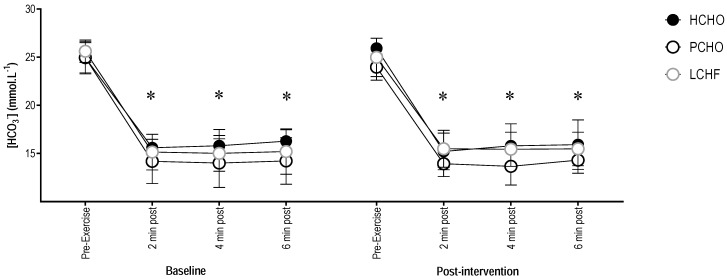
Blood [HCO^3−^] for the high carbohydrate (HCHO), periodized carbohydrate (PCHO), and low carbohydrate high fat (LCHF) groups for baseline and post-testing at pre-exercise, plus 2, 4, and 6 min post-test. * Significantly different to pre-exercise (*p* < 0.001).

There were no significant differences in pre-exercise or post-exercise blood [HCO_3_^−^] between groups at baseline (F(2,20.99) = 3.31; *p* = 0.06), or post-training intervention (F(1,161.15) = 0.14; *p* = 0.71). As with blood pH, there was a significant decrease in blood [HCO_3_^−^] (95% CI = (−10.74; −9.53)) between pre-exercise and all post-exercise timepoints for HCHO, PCHO, and LCHF (F(1,161.16) = 511.24; *p* < 0.001) (Figure 3).

### 3.4. Blood Lactate Concentration

**Figure 4:**
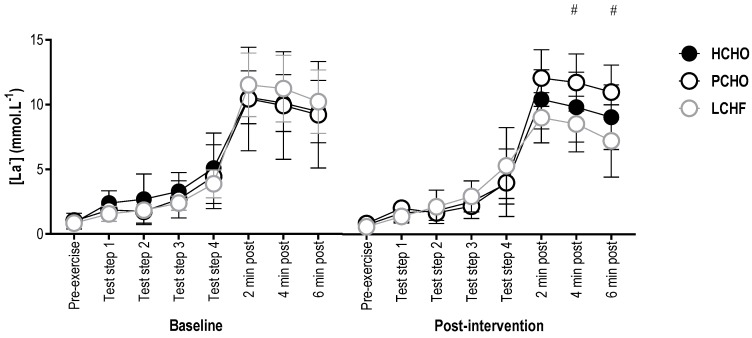
Blood [La^−^] for the high carbohydrate (HCHO), periodized carbohydrate (PCHO) and low carbohydrate high fat (LCHF) groups for baseline and post-testing at pre-exercise, steps 1, 2, 3, and 4 of the economy test, and 2, 4, and 6 min post-test. # PCHO significantly different to LCHF (*p* < 0.05).

There were no significant differences in blood [La^−^] between groups at baseline for any timepoint (F(2,20.98) = 0.96, *p* = 0.40). Post-intervention, there were no significant differences between groups (F(14,311.07) = 1.28; *p* = 0.22) for pre-exercise blood [La^−^] (95% CI = (−2.15; 2.07) and (−2.14; 2.19) for LCHF and HCHO, compared to PCHO, respectively), or at any stage during the economy test (95% CI = (−2.22; 3.53) and (−2.13; 4.00) for LCHF and PCHO, compared to HCHO, respectively), or 2 min post-test (95% CI = (−5.13; 0.62) and (−1.27; 4.86) for LCHF and PCHO, compared to HCHO, respectively). At 4 min post-test, blood [La^−^] was significantly higher for PCHO than LCHF (95% CI = (1.52; 7.57)), with no difference between PCHO and HCHO control (95% CI = (−5.12; 1.07)). Similarly, blood [La^−^] was significantly higher 6 min post-exercise with PCHO compared with LCHF (95% CI = (1.77; 7.74)), and there was no difference compared with HCHO (95% CI = (−5.27; 0.86)) (Figure 4).

### 3.5. Net Endogenous Acid Production

There were significant differences between NEAP_F1_, NEAP_F2_, and NEAP_R_ (F(2,115) = 39.02, *p* < 0.001). For all groups and at both baseline and post-intervention, NEAP_F1_ values were significantly higher compared to NEAP_R_ (95% CI = (17.40; 30.44)), with no significant difference between NEAP_F2_ and NEAP_R_ (95% CI = (−9.90; 3.13)) (Table 3). At baseline, NEAP was lower for HCHO compared to LCHF (95% CI = (−28.22; −3.54)), and there was no difference between PCHO and LCHF (95% CI = (−20.82; 4.77)). Post-intervention, NEAP values remained unchanged for LCHF (95% CI = (−6.91; 10.47)), but there was a significant decrease for the HCHO (95% CI = (−20.70; −227)) and PCHO (95% CI = (−23.29; −3.58)). Post-intervention, NEAP was significantly higher in LCHF compared with HCHO control (95% CI = (10.44; 36.04)) but there was no difference for PCHO compared with HCHO (95% CI = (−19.06; 7.23)).

## 4. Discussion

This is the first study to investigate the effects of sustained manipulation of macronutrient intake on acid–base status in elite athletes. The main finding of this investigation was that substantial restriction of dietary carbohydrate intake with a ketogenic low carbohydrate high fat diet over a three-week period had no influence on resting or exercise-associated blood indices of acid–base status, despite changes in the calculated net endogenous acid production of the diet. While it is plausible that this may be an anomaly that is specific to the training status and its effect on muscle buffering capacity of elite athletes in this study, it may also reflect the neutralization or expulsion of dietary acids through the respiratory or renal pathways. 

The findings of this study contrast with earlier investigations, where low carbohydrate high fat diets were associated with decreased resting blood pH and blood [HCO_3_^−^] [15,16,17]. However, there are several important differences between our study and those conducted previously. Our study involved elite athletes, most of whom had represented their countries at World Championships, Olympic Games, and International Association of Athletics Federations (IAAF) World Cup events, whereas the participants in previous studies were healthy but untrained [15,16,17]. Furthermore, the dietary intervention was applied for 21 days in the present investigation, in comparison to acute (three or four-day) interventions [15,16,17], potentially affording sufficient time for acids to be neutralized by blood and tissue buffer systems, or eliminated through the respiratory or renal systems [25]. Finally, unlike previous studies, a high level of control was imposed in the current investigation, with dietary intake being individually prescribed and consumed under supervision of qualified dietitians [32]. 

Race walking competitions require sustained efforts at high velocities [41], and athletes maintain a high percentage of maximal heart rate throughout races [42]. High-intensity training sessions are an important component of race walkers’ training programs [42,43], and include 1 km (~4 min) and 2 km (~8.5 min) repetitions performed close to race pace, plus hill sessions [43]. Indeed, evidence of the intensity of several of the weekly training sessions in the current study was provided by observations of post-exercise blood lactate concentrations as high as 19.2 mmol·L^−1^ after the prescribed session of 1 km repetitions. Such training sessions are likely to contribute to highly adapted buffering capacities, particularly within skeletal muscle, among the race walkers in this study [44,45]. Indeed, the buffering capacity in athletes is typically more adapted than in untrained individuals [32,33], which may explain why we did not observe any post-intervention differences in resting acid–base status between participants in the LCHF and PCHO or HCHO groups, despite elevations in ketone body concentration (typically sustained at ~1 mmol·L^−1^) in the LCHF group (Burke et al.; submitted for publication). Furthermore, it is likely that the well-developed buffering capacity of the athletes was evident prior to the start of the study, rather than specifically improving over the course of the study as a result of the intensified training that was performed, given that no differences between baseline and post-intervention sub-maximal exercise blood lactate concentrations were observed. There is some evidence from other approaches to modifying acid–base status in elite athletes that buffering capacity is already robust, and perhaps already able to cope with their regular exposure to perturbations associated with high-intensity exercise. Indeed, among the few studies specifically examining responses to supplementation with extra-cellular buffering agents, such as bicarbonate or citrate, in elite athletes across different exercise modalities [46,47], the improvements in exercise capacity or performance normally observed in modestly trained or lower caliber athletes [48] are not detected.

While some differences in post-exercise blood pH were evident as a consequence of sustained macronutrient manipulation, this may merely reflect differences in work capacity. Specifically, post-exercise blood pH was lower, while blood [La^−^] was higher following PCHO, compared with LCHF. However, the PCHO group in this study performed more work during the post-intervention graded exercise test, with a mean test duration of 17.6 min for PCHO, compared with 15.8 min for LCHF, and race performance was impaired in the LCHF group (Burke et al., submitted for publication). Similar suppression of blood [La^−^] associated with impaired work capacity has been reported in elite athletes of a similar caliber and training status to the current study [9].

In this study, we observed a higher post-intervention NEAP with LCHF, compared with both PCHO and HCHO. The fact that a similar trend was not observed in either post-intervention blood bicarbonate concentration or blood pH, despite evidence of increased ketone body concentration (Burke et al.; submitted for publication), which can contribute to acidosis [4] in the LCHF group, may provide indirect evidence of well-developed blood buffering capacity of the elite athletes in the present study. This observation suggests that NEAP calculations may have little applicability to highly trained athletes. 

One limitation of this study is that the results are specific to our participant population, where the sample size was limited both by the resources needed to undertake the investigation and, by the definition of elite, to a small sub-group with special characteristics. Future research is warranted, whereby NEAP and direct measures of acid–base status are monitored after dietary interventions in different participant populations, with a similar level of rigor applied to the dietary control to that implemented in the current investigation. A controlled environment, such as that involved in the current investigation may lead to an alteration in dietary intake from usual choices [49], and an increase in the day-to-day variability of NEAP. Indeed, the self-chosen diets at baseline in the present study showed a greater range than the prescribed diets, even when consumed from the same menu options. Nevertheless in terms of adequately testing hypotheses around this topic, dietary standardization is important [49]. A further limitation of the current investigation was that 24 h urinary net acid excretion (NAE) was not included in the measures taken. It is acknowledged that the collection of NAE allows more accurate estimations of NEAP than equations based on dietary constituents [50], however, it was not feasible to collect 24 h urine samples in the current study. Future investigations may incorporate the direct quantification of NEAP via the measurement of NAE, or if resources are constrained, 24 h pH may be investigated since it reasonably correlates with, and explains the variability in, 24 h NAE [27,51]. 

## 5. Conclusions

Our results indicate that sustained manipulation of macronutrient intake is unlikely to influence acid–base status in elite athletes. Thus, any performance implications of a ketogenic low carbohydrate high fat diet are unlikely to result from perturbations in acid–base status, a system which is tightly regulated via the interaction of the blood and tissues, plus respiratory and renal systems. Finally, the implications of sustained manipulation of dietary carbohydrate on NEAP may be best assessed via the criterion 24 h urinary net acid excretion (NAE) method, over indirect NEAP calculations, in highly trained athletes. 

## Figures and Tables

**Table 1:** Participant characteristics (mean ± SD). Nutritional intake is presented as daily intake relative to body mass (BM), and as a percentage of total energy intake.

	Males (*n* = 18)	Females (*n* = 7)
**BASELINE TEST RESULTS**		
**Height (cm)**	178.4 ±. 6.3	165.9 ± 5.8
**Mass (kg)**	67.7 ± 5.4	54.1 ± 5.1
**VO_2_max (mL·kg·min^−1^)**	60.5 ± 4.5	56.2 ± 3.6
**BASELINE NUTRITIONAL INTAKE**		
**Carbohydrate (g·kg^−1^ BM)**	6.6 ± 1.3 (53 ± 7%)	7.1 ± 1.5 (53 ± 9%)
**Protein (g·kg^−1^ BM)**	2.5 ± 0.6 (20 ± 4%)	2.5 ± 0.6 (18 ± 4%)
**Fat (g·kg^−1^ BM)**	1.5± 0.5 (26 ± 5%)	1.6 ± 0.4 (26 ± 5%)

**Table 2:** Template for weekly training program. Shading indicates mandatory sessions; remaining sessions could be modified by individual athletes.

DAY	Monday	Tuesday	Wednesday	Thursday	Friday	Saturday	Sunday
AM	10 km walk	10–15 km walk ^#^ Resistance training	>20 km long walk Hydrotherapy strategies	10 km walk Resistance training	Hill session ^†^	>20 km long walk Hydrotherapy strategies	10 km walk or rest
PM	1 km reps ^†^ Hydrotherapy strategies	10 km walk	10 km walk or rest	10–15 km walk	10–15 km walk ^#^	10 km walk or rest	

^#^ Indicates training session with low carbohydrate availability prior to the session (PCHO group only). ^†^ Indicates training session with low carbohydrate availability after the session (PCHO group only).

**Table 3:** Net endogenous acid production (NEAP) estimations for high carbohydrate (HCHO), periodized carbohydrate (PCHO) and low carbohydrate, high fat (LCHF) groups using two equations by Frassetto et al. (NEAP_F1_, NEAP_F2_) [25], and one equation by Remer and Manz (NEAP_R_) [26]. NEAP estimations are provided for baseline and post-testing.

	HCHO	PCHO	LCHF
**BASELINE**			
**NEAP_F1_ (mEq·day^−1^)**	75 ± 19	84 ± 18	104 ± 35
**NEAP_F2_ (mEq·day^−1^)**	49 ± 9	62 ± 16	61 ± 15
**NEAP_R_ (mEq·day^−1^)**	62 ± 19 *	63 ± 14 *	68 ± 31 *
**POST-INTERVENTION**			
**NEAP_F1_ (mEq·day^−1^)**	65 ± 12	71 ± 16	98 ± 15
**NEAP_F2_ (mEq·day^−1^)**	45 ± 4	48 ± 4	70 ± 7
**NEAP_R_ (mEq·day^−1^)**	40 ± 10 *	58 ± 35 *	69 ± 17 *

* significantly different to NEAP_F1_ (*p* < 0.05).

## Supplementary Materials

The Supplementary Materials are available online at http://www.mdpi.com/2072-6643/10/2/236/s1.
