# Supplementary material for: Chronic Ketogenic Low Carbohydrate High Fat Diet Has Minimal Effects on Acid–Base Status in Elite Athletes
Source: Nutrients. 2018 Feb 18;10(2):236. doi: 10.3390/nu10020236 (PMC5852812; doi:10.3390/nu10020236)
Supplement: Supplementary file 1 [file nutrients-10-00236-s001.pdf]

Supplementary Table S1: Example of a weekly menu for the low carbohydrate, high fat (LCHF) diet.

**Time**	**Monday**	**Tuesday**	**Wednesday**	**Thursday**	**Friday**	**Saturday**	**Sunday**
**Pre-training**	Peanut Butter cookies<br>Macadamia nuts	Granola<br>Edam cheese	Egg<br>Cheese<br>Chocolate pecan cake<br>Electrolytes/diet coke		Low carb bread, butter & cheese	Egg<br>Cheese<br>Chocolate pecan cake<br>Electrolytes/diet coke	
**Breakfast**	Granola<br>Greek yoghurt<br>Almond milk	Savory crepe<br>Bacon<br>Avocado		Berry & coconut parfait	Cauliflower rösti<br>Egg<br>Hollandaise sauce		Bircher muesli
**Lunch**	Caesar salad	Turkey & brie sandwich	Fried caulirice<br>Egg<br>Bacon	Silverside, tomato, cheese & avocado sandwich	Mushroom soup	Frittata<br>Avocado<br>Green salad	Chicken, pesto, cheese & salad sandwich
**Dinner**	Lamb satay<br>Avocado<br>Green salad<br>Olive oil	Carbonara with zucchini noodles<br>Green salad<br>Olive oil<br>Choc coconut ice-cream	Chicken curry<br>Whipped cream<br>Berries	Beef taco salad<br>Crème fraiche<br>Green salad	Salmon<br>Cauliflower risotto<br>Green salad<br>Berry tiramisu	Beef burgers with low carb bread<br>Chocolate cake with cream	Pizza with cauliflower base<br>Havarti cheese<br>Green salad<br>Olive oil
**Snacks**	Peanut butter cookies<br>Macadamia nuts	Lemon fat bomb<br>Granola<br>Edam cheese	Vanilla cookie-dough amaze bites<br>Brie cheese<br>Almonds	Nut balls<br>Buttered coffee<br>Choc and pecan cake	Choc pecan cake<br>Chocolate peanut fat bomb<br>Low CHO bread & edam cheese	Peanut butter cookies<br>Vanilla cookie-dough amazebites<br>Brie	Peanut butter cookies<br>Low CHO bread & brie<br>Chunky nut cookies
**Recovery**	Recovery drink & whey protein	Recovery Drink	Recovery Drink	Double chocolate shake		Recovery drink	

Table adapted with permission from Mirtschin, J.; Forbes, S.; Cato, L.; Heikura, I.; Strobel, N.; Hall, R.; Burke, L. Organisation of dietary control for training-nutrition intervention involving low carbohydrate high fat (LCHF) diet. *Int. J. Sport Nutr. Exerc. Metab.* (In Press).

Supplementary Table S2: Example of a weekly menu for the periodized carbohydrate availability (PCHO) diet.

Time	Monday	Tuesday	Wednesday	Thursday	Friday	Saturday	Sunday
**Pre-training**	Gatorade		White bread, honey & butter<br>Juice<br>Gatorade/gel/ lollies		White bread, jam & butter<br>Energy gel<br>Gatorade	White bread, jam & butter<br>Juice<br>Gatorade/gel/ lollies	
**Breakfast**	Vita-brits<br>Milk<br>Banana<br>Honey/sugar	Pancakes<br>Berries<br>Maple syrup<br>Greek yoghurt		Cereal<br>Skim milk<br>White bread & jam	Omelette<br>Bagel<br>Spreads		Bircher muesli<br>Fruit
**Lunch**	Ham & salad wrap<br>Fruit<br>Yoghurt	Turkey, cranberry & salad sandwich<br>Yoghurt	Fried Rice<br>Yoghurt	Silverside, tomato & cheese sandwich<br>Yoghurt	Potato soup<br>Bacon<br>Bread<br>Fruit	Frittata<br>Fruit	Chicken, pesto & salad sandwich<br>Juice
**Dinner**	Chicken stir fry<br>Salad	Pasta with Bolognese sauce<br>Cheese<br>Salad<br>Ice cream & chocolate syrup	Chicken curry<br>Rice<br>Fruit<br>Greek yoghurt<br>Honey	Mexican mince burritos<br>Salad<br>Crème fraiche	Salmon<br>Risotto<br>Salad<br>Juice<br>Fruit & custard	Beef burger<br>Banana bread	Pizza
**Snacks**	Date balls<br>Chocolate milk<br>Macadamia nuts<br>Rice bar	Rice cakes & peanut butter/jam<br>Dried fruit<br>Skim milk	Nut balls<br>Fresh fruit<br>Chocolate milk<br>Yoghurt	Rice cakes & peanut butter /jam<br>Banana<br>Yoghurt<br>Dried fruit	Rice bar<br>Muesli bar<br>Fresh Fruit<br>Cashews	Rice cakes & peanut butter/jam<br>Fresh fruit<br>Date balls<br>Yoghurt	Date balls<br>Yoghurt<br>Fruit<br>Nut balls
**Recovery**	Whey protein & water	Banana & berry smoothie	Sustagen recovery drink	Banana & berry smoothie	Whey protein & milk	Sustagen recovery drink	

Table adapted with permission from Mirtschin, J.; Forbes, S.; Cato, L.; Heikura, I.; Strobel, N.; Hall, R.; Burke, L. Organisation of dietary control for training-nutrition intervention involving low carbohydrate high fat (LCHF) diet. *Int. J. Sport Nutr. Exerc. Metab.* (In Press).

Supplementary Table S3: Example of a weekly menu for the high carbohydrate availability (HCHO) control group diet.

Time	Monday	Tuesday	Wednesday	Thursday	Friday	Saturday	Sunday
**Pre-training**	White bread, butter & jam<br>Gatorade	Energy gel<br>Muesli bar<br>Gatorade	White bread, honey & butter<br>Juice<br>Gatorade/gel/lollies	Energy gel<br>Muesli bar<br>Gatorade	Energy gel<br>Muesli bar<br>Gatorade	White bread, jam & butter<br>Juice<br>Gatorade/gel/lollies	
**Breakfast**	Vita-brits<br>Milk<br>Banana<br>Honey/sugar	Pancakes<br>Berries<br>Maple syrup<br>Greek yoghurt		Cereal<br>Skim milk<br>White bread & jam	Omelette<br>Bagel<br>Spreads		Bircher muesli<br>Fruit
**Lunch**	Ham & salad wrap<br>Fruit<br>Yoghurt	Turkey, cranberry & salad sandwich<br>Yoghurt	Fried rice<br>Yoghurt	Silverside, tomato & cheese sandwich<br>Yoghurt	Potato soup<br>Bacon<br>Bread<br>Fruit	Frittata<br>Fruit	Chicken, pesto & salad sandwich<br>Juice
**Dinner**	Lamb stir fry<br>Rice	Pasta with Bolognese sauce<br>Cheese<br>Salad<br>Ice cream & chocolate syrup	Chicken curry<br>Rice<br>Fruit<br>Greek yoghurt<br>Honey	Mexican mince burritos<br>Salad<br>Crème fraiche	Salmon risotto<br>Salad<br>Juice<br>Fruit & custard	Beef burger<br>Banana bread	Pizza
**Snacks**	Date balls<br>Chocolate milk<br>Macadamia nuts<br>Rice bar	Rice cakes & peanut butter/jam<br>Dried fruit<br>Skim milk	Nut balls<br>Fresh Fruit<br>Chocolate milk<br>Yoghurt	Rice cakes & peanut butter /jam<br>Banana<br>Yoghurt<br>Dried fruit	Rice bar<br>Muesli bar<br>Fresh fruit<br>Cashews	Rice cakes & peanut butter/jam<br>Fresh fruit<br>Date balls<br>Yoghurt	Date balls<br>Yoghurt<br>Fruit<br>Nut balls
**Recovery**	Sustagen recovery drink	Banana & berry smoothie	Sustagen recovery drink	Banana & berry smoothie	Whey protein & milk	Sustagen recovery drink	

Table adapted with permission from Mirtschin, J.; Forbes, S.; Cato, L.; Heikura, I.; Strobel, N.; Hall, R.; Burke, L. Organisation of dietary control for training-nutrition intervention involving low carbohydrate high fat (LCHF) diet. *Int. J. Sport Nutr. Exerc. Metab.* (In Press).
